# Active Compensation Fault-Tolerant Control for Uncertain Systems with Both Actuator and Sensor Faults

**DOI:** 10.3390/s26010267

**Published:** 2026-01-01

**Authors:** Xufeng Ling, Haichuan Xu, Fanglai Zhu

**Affiliations:** 1 School of Artificial Intelligence, Shanghai Normal University Tianhua College, Shanghai 201815, China; lxf1131@sthu.edu.cn; 2 Shanghai Research Institute for Intelligent Autonomous Systems, Tongji University, Shanghai 200092, China; xuhaichuan@tongji.edu.cn; 3 School of Electronics and Information Engineering, Tongji University, Shanghai 201804, China

**Keywords:** actuator and sensor faults, fault reconstruction, reduced-order observer, interval observer, multiple unknown input

## Abstract

This paper develops a novel fault reconstruction (FR) method and an FR-based fault-tolerant control (FTC) scheme for systems suffering from both sensor and actuator faults based on the combination of a Luenberger-like reduced-order observer and an interval observer. Firstly, by introducing an output transformation, an auxiliary output that is able to decouple the sensor fault is obtained. Secondly, for addressing the external disturbance and actuator fault, a multiple unknown input (MUI) is formed, and a reduced-order observer that is able to decouple the MUI is constructed. Consequently, asymptotic convergence estimations of the state and the sensor fault can be accomplished. Thirdly, in order to obtain the asymptotic convergence actual FR (AFR), an interval observer is designed. After this, an algebraic connection of the MUI and the state error estimation is given, and, based on the algebraic relationship, an algebraic MUI reconstruction (MUIR) method is proposed. Finally, an FTC scheme is developed by using the state estimation and MUIR. Under the FTC, the closed-loop system is asymptotically stable even if it suffers from sensor and actuator faults simultaneously. Theoretical analysis demonstrates that the observer-based FTC mechanism satisfies the separation principle. At last, two simulation examples are given to verify the effectiveness of the proposed methods.

## 1. Introduction

Ensuring the reliability and safety of practical control system operations is always a critical and challenging issue in control theory and control engineering. Actuators and sensors, as essential components, are particularly vulnerable to malfunctions due to their frequent operations in harsh environments [1,2,3,4,5,6], resulting in faults which may greatly degrade system performances. Therefore, the techniques of fault detection (FD), fault reconstruction (FR), and fault-tolerant control (FTC) play crucial roles in maintaining system performances when systems suffer from actuator and sensor faults. Hence, the FD, FR, and FTC techniques have been investigated intensively for decades and many significant methods have been developed for varieties of control systems [7,8,9,10,11,12,13,14,15,16,17,18,19]. For instance, in [11], a distributed adaptive event-triggered FTC scheme was proposed with a hierarchical structure for heterogeneous multi-agent systems (MASs), which prevents fault propagation and eliminates the need for continuous communication between agents. In [12], FDI of an active suspension system with actuator and sensor faults was investigated with two unknown input observers. FR can let the designers know more information about systems and, thus, make the designs much more convenient. Many researchers have explored FR techniques in the context of various control systems [13,14,15,16]. In [17], an intelligent-observer-based precise and fast AFR approach was developed. In [18], a fuzzy synthesized learning and Luenberger-like observer method was proposed for AFR in T-S fuzzy systems. In [19], for a nonlinear descriptor system, a robust AFR was designed based on adaptive observer for addressing the actuator additive faults.

Generally speaking, FTC techniques can be divided into two main categories: active fault-tolerant control (AFTC) and passive fault-tolerant control (PFTC). PFTC can be regarded as a robust control scheme constructed to reject the fault, without considering the fault occurrences or forms. On one hand, although the flexibility of a PFTC is lower than that of an AFTC in dealing with the faults, research on PFTC can also be found in the literature [20,21,22,23,24,25]. On the other hand, the AFTC method becomes the dominated FTC because of its flexibility and high pertinence to faults. The major strategy of an AFTC is adjusting the controller by referring to fault conditions, resulting in a better fault-tolerance performance. It should be emphasized that observer-based FR has become one of the fundamentals of AFTC. Therefore, in recent years, the AFTC methods have gained much attention [26,27,28,29,30,31]. For instance, a dynamic output feedback FTC strategy for switched affine systems subject to actuator faults was proposed, utilizing fault estimation and a mode-dependent dwell-time switching scheme to ensure practical exponential stability [32]. Similarly, an observer-based robust FTC scheme was proposed for discrete-time T-S fuzzy models with unavailable premise variables [33]. Kalman-filter-based fault detection and diagnosis were investigated for fault-tolerant control in quadcopters, improving safety under sensor and actuator faults in [34]. By leveraging model-based strategies, these approaches enable systems to maintain functionality even under fault conditions, underscoring the practical advantages of observer-based FTC in real-world applications.

Most existing work on observer-based FD, FR, or FTC focuses on dealing with either actuator or sensor faults separately [35,36]; little work can be found for handling actuator and sensor faults simultaneously [37,38,39,40]. In the present paper, FR and FTC design problems are discussed for uncertain systems subject to both actuator and sensor faults, as well as external disturbances. The major contributions of the paper are summarized as follows:

**(1) Novel Integrated Unified Framework.** Most existing observer-based FTC studies focus on handling either actuator faults or sensor faults separately. This work tackles a more challenging and realistic scenario where actuator faults, sensor faults, and external disturbances coexist in the system. The proposed unified FR and FTC framework provides a systematic solution for concurrent multi-fault situations for uncertain systems.

**(2) MUI Construction and Asymptotic Reconstruction.** The actuator fault, external disturbance, and the control input are aggregated into a single MUI. A novel MUIR scheme is developed via an interval observer that provides asymptotic estimates of the actual MUI. Crucially, the proposed MUIR decouples the control signal, and this significant decoupling feature is essential for the subsequent controller synthesis.

**(3) Separation-Principle-Based FTC Design.** The FTC framework consists of a reduced-order observer, an MUIR, and a state-feedback controller. We point out that the three parts satisfy the so-called separation principle. Therefore, a convenient design procedure of observer-based FTC for handling both actuator and sensor faults simultaneously is developed.

The remainders of this paper are organized as follows: Section 2 introduces the preliminaries. Section 3 designs a reduced-order observer for obtaining the estimation of the state and sensor fault reconstruction (SFR). Section 4 designs an interval observer for reconstructing MUIs, and Section 5 verifies the effectiveness of the proposed methods by two simulation examples. Section 6 provides the conclusions of the paper.

## 2. Preliminaries

In this section, some preliminaries, including notations, lemmas, system description, and preconditions, are presented.

**Notations:** $S=\left[s_{ij}\right]\in R^{n\times m}$ is an n×m matrix. We denote $S^{+}=\left[s_{ij}^{+}\right],S^{-}=\left[s_{ij}^{-}\right]$ and $\left|S\right|=\left[\left|s_{ij}\right|\right]$, where $s_{ij}^{+}=\max\left\{0,s_{ij}\right\}$ and $s_{ij}^{-}=\max\left\{0,-s_{ij}\right\}$. Then, obviously, we have $S=S^{+}-S^{-}$ and $\left|S\right|=S^{+}+S^{-}$. For another matrix $R=\left[r_{ij}\right]\in R^{n\times m},S\leq R\Leftrightarrow s_{ij}\leq r_{ij}\;\left(i=1,\cdots ,n;j=1,\cdots ,m\right)$. $0_{m}\in R^{m}$ and $1_{m}\in R^{m}$ denote all-zero and all-one column vectors, respectively.

**Definition** **1.** *A matrix is a Metzler matrix if all of its off-diagonal entries are nonnegative. A matrix is a Hurwitz matrix if all of its eigenvalues have negative real parts.*

**Lemma** **1** ([10]). *For any matrix $S\in R^{m\times n}$, one has $S^{+}\underline{m}\left(t\right)-S^{-}\bar{m}\left(t\right)\leq Sm\left(t\right)\leq S^{+}\bar{m}\left(t\right)-S^{-}\underline{m}\left(t\right)$, if $\underline{m}\left(t\right)\leq m\left(t\right)\leq \bar{m}\left(t\right)$, where $\underline{m}\left(t\right)\in R^{n}$, $m\left(t\right)\in R^{n}$ and $\bar{m}\left(t\right)\in R^{n}$.*

**Lemma** **2** ([41]). *For the system $\dot{x}\left(t\right)=Ax\left(t\right)+\rho \left(t\right)$, one has x(t)≥0 for t≥0, if A is a Metzler matrix, ρ(t)≥0 and x(0)≥0.*

Consider system

(1) $$\begin{array}{c} \left\{\begin{array}{cc} \dot{x}\left(t\right) & =Ax\left(t\right)+B\left(u_{f}\left(t\right)+d\left(t\right)\right)\\ y(t) & =Cx\left(t\right)+Ff_{s}\left(t\right) \end{array}\right. \end{array}$$

where $x\left(t\right)\in R^{n}$ and $y\left(t\right)\in R^{p}$ are system state and output vectors, respectively. $d\left(t\right)\in R^{m}$ is the external disturbance impacting on the system state equation. In addition, $f_{s}\left(t\right)\in R^{r}$ stands for the sensor faults which need to be reconstructed. $u_{f}\left(t\right)\in R^{m}$ stands for the output of the actuator which can be modeled as $u_{f}\left(t\right)=\rho \left(t\right)u\left(t\right)+f_{a}\left(t\right)$, where ρ(t)∈R and $f_{a}\left(t\right)\in R^{m}$ are the efficiency factor and bias signal of the actuator fault, respectively. When ρ(t)=1 and $f_{a}\left(t\right)=0$, the actuator is in a healthy condition; otherwise, it is in a faulty condition. Thereby, the original system (1) can be modeled as

(2) $$\begin{array}{c} \left\{\begin{array}{cc} \dot{x}\left(t\right) & =Ax(t)+B(u(t)+w(t))\\ y(t) & =Cx\left(t\right)+Ff_{s}\left(t\right) \end{array}\right. \end{array}$$

where u(t) is the actuator control input that needs to be designed, and $w\left(t\right)=\left(\rho \left(t\right)-1\right)u\left(t\right)+f_{a}\left(t\right)+d\left(t\right)$ can be taken as a multiple unknown input (MUI) of (2).

**Assumption** **1.** *The following rank condition:*

(3) $$\begin{array}{c} \mathrm{rank}\left[\begin{array}{ccc} sI_{n}-A & B & 0\\ C & 0 & F \end{array}\right]=n+m+r \end{array}$$

*is true for all complex scalar s with Re(s)≥0.*

**Assumption** **2.** *The rank condition*

(4) $$\begin{array}{c} \mathrm{rank}\left[\begin{array}{ccc} I_{n} & B & 0\\ C & 0 & F \end{array}\right]=n+m+r \end{array}$$

*holds.*

**Assumption** **3.** *The actuator control input u(t), efficiency factor ρ(t), bias signal $f_{a}\left(t\right)$, external disturbance d(t), and initial state x(0) are all bounded with $\underline{u}\leq u\left(t\right)\leq \bar{u},\underline{d}\leq d\left(t\right)\leq \bar{d},{\underline{x}}_{0}\leq x\left(0\right)\leq {\bar{x}}_{0},0\leq \underline{\rho }\leq \rho \left(t\right)\leq \bar{\rho }\leq 1$ and ${\underline{f}}_{a}\leq f_{a}\left(t\right)\leq {\bar{f}}_{a}$, where $\underline{u},\bar{u},\underline{d},\bar{d},{\underline{x}}_{0},{\bar{x}}_{0},{\underline{f}}_{a}$ and ${\bar{f}}_{a}$, are known constant vectors, and $\underline{\rho }$ and $\bar{\rho }$ are two known constant scalars. The sensor fault $f_{s}$ is bounded.*

Obviously, under Assumption 3, we have $\underline{w}\leq w\leq \bar{w}$, where $\underline{w}=\underline{\rho }\underline{u}-\bar{u}+\underline{f_{a}}+\underline{d}$ and $\bar{w}=\bar{\rho }\bar{u}-\underline{u}+{\bar{f}}_{a}+\bar{d}$.

**Remark** **1.** *In our method, all kinds of faults described by Table 1 can be handled in a general way: by constructing an MUI $w\left(t\right)=\left(\rho \left(t\right)-1\right)u\left(t\right)+f_{a}\left(t\right)+d\left(t\right)$. The major technique we used is developing an MUIR that can estimate the actual MUI w(t) asymptotically in advance, and then designing a compensation controller by introducing the MUIR into the controller, leading to the closed-loop system under the controller being asymptotically stable. It should be noted that when ρ(t)≠1, the MUI w(t) contains the control input u(t) which needs to be designed. In other words, the MUI couples with the control signal. Therefore, to accomplish the design goal by these two steps is a really challenging task because of the coupling between w(t) and u(t).*

## 3. Reduced-Order Observer and SFR

In this section, an output transformation is made and an auxiliary output which decouples the sensor fault is obtained. Then, a reduced-order observer which is able to estimate the state and reconstruct the sensor fault is designed.

### 3.1. Output Transformation

Make an output transformation of $\tilde{y}=\left(I_{p}-FF^{†}\right)y$, then the system (1) or (2) can be expressed by

(5) $$\begin{array}{c} \left\{\begin{array}{cc} \dot{x} & =Ax+B(u+w)\\ \tilde{y} & =\tilde{C}x \end{array}\right. \end{array}$$

where $\tilde{y}=\tilde{C}x$ is an auxiliary output with $\tilde{C}=\left(I_{p}-FF^{†}\right)C$. Here, we denote by $F^{†}$ the generalized inverse of *F* satisfying $FF^{†}F=F$, and when *F* is of full column rank, it has the exact expression of $F^{†}={\left(F^{T}F\right)}^{-1}F^{T}$.

**Lemma** **3.** *When the sensor suffers from a fault, i.e., when r>0, we have $\mathrm{rank}\left(\tilde{C}\right)=p-r:=\overset{⌢}{p}<p$, which indicates that matrix $\tilde{C}$ is not a full row matrix.*

**Proof.**  On one hand, note that matrix *F* is with full column rank, then according to the singular value decomposition (SVD) method, there exist two orthogonal matrices $Y\in R^{p\times p}$ and $Z\in R^{r\times r}$ such that $F=Y\left[\begin{array}{c} \Sigma \\ 0_{\overset{⌢}{p}\times r} \end{array}\right]Z^{T}$, where $\Sigma =\mathrm{diag}\left(\sigma_{1},\cdots ,\sigma_{r}\right)$ with $\sigma_{1},\cdots ,\sigma_{r}$ being singular values of matrix *F*. On the other hand, note that $F^{†}={\left(F^{T}F\right)}^{-1}F^{T}$; then, by computing directly, we have

$$F^{†}=Z\left[\begin{array}{cc} \Sigma^{-1} & 0_{r\times \overset{⌢}{p}} \end{array}\right]Y^{T}$$

and

$$FF^{†}=Y\left[\begin{array}{cc} I_{r} & 0_{r\times \overset{⌢}{p}}\\ 0_{\overset{⌢}{p}\times r} & 0_{\overset{⌢}{p}\times \overset{⌢}{p}} \end{array}\right]Y^{T}$$

Thus,

$$\tilde{C}=\left(I_{p}-FF^{†}\right)C=\left(YY^{T}-Y\left[\begin{array}{cc} I_{r} & \\ & 0_{\overset{⌢}{p}\times \overset{⌢}{p}} \end{array}\right]Y^{T}\right)C=Y\left[\begin{array}{cc} 0_{r\times r} & \\ & I_{\overset{⌢}{p}} \end{array}\right]Y^{T}C$$

which implies that $\mathrm{rank}\left(\tilde{C}\right)=\overset{⌢}{p}$. □

Now, from Lemma 3, we know that $\tilde{C}$ does not have full row rank, then there exists a nonsingular matrix $\Upsilon \in R^{p\times p}$ such that $\Upsilon \tilde{C}=\left[\begin{array}{c} \overset{⌢}{C}\\ 0_{r\times n} \end{array}\right]$, where $\overset{⌢}{C}\in R^{\overset{⌢}{p}\times n}$ is a matrix which is formed by the maximal linearly independent row vectors of $\tilde{C}$, and naturally $\mathrm{rank}\left(\overset{⌢}{C}\right)=\overset{⌢}{p}$, i.e., $\overset{⌢}{C}$ has full row rank. Obviously, it holds that $\overset{⌢}{C}=\left[\begin{array}{cc} I_{\overset{⌢}{p}} & 0_{\overset{⌢}{p}\times r} \end{array}\right]\Upsilon \tilde{C}=\Phi C$, where $\Phi =\left[\begin{array}{cc} I_{\overset{⌢}{p}} & 0_{\overset{⌢}{p}\times r} \end{array}\right]\Upsilon \left(I_{p}-FF^{†}\right)\in R^{\overset{⌢}{p}\times p}$. If we introduce once more the auxiliary output of $\overset{⌢}{y}=\Phi y$, then we have $\overset{⌢}{y}=\overset{⌢}{C}x$. As a result, System (1) is then further rewritten as
  (6) $$\begin{array}{c} \left\{\begin{array}{cc} \dot{x} & =Ax+B(u+w)\\ \overset{⌢}{y} & =\overset{⌢}{C}x \end{array}\right. \end{array}$$
  with $\overset{⌢}{C}$ being of full row rank.

**Lemma** **4.** *Under Assumption 1, the rank condition*

(7) $$\begin{array}{cc} \mathrm{rank}\left[\begin{array}{cc} sI_{n}-A & B\\ \overset{⌢}{C} & 0 \end{array}\right] & =\mathrm{rank}\left[\begin{array}{cc} sI_{n}-A & B\\ \tilde{C} & 0 \end{array}\right]=n+m \end{array}$$

*is true for all complex scalar s with Re(s)≥0.*

**Proof.**  Based on (3) in Assumption 1, we can deduce that

$$\begin{array}{cc} n+m+r & =\mathrm{rank}\left[\begin{array}{ccc} sI_{n}-A & B & 0\\ C & 0 & F \end{array}\right]\\ \; & =\mathrm{rank}\left\{\left[\begin{array}{ccc} sI_{n}-A & B & 0\\ C & 0 & F \end{array}\right]\left[\begin{array}{ccc} I_{n} & 0 & 0\\ 0 & I_{q} & 0\\ -F^{†}C & 0 & I_{r} \end{array}\right]\right\}\\ \; & =\mathrm{rank}\left[\begin{array}{ccc} sI_{n}-A & B & 0\\ \tilde{C} & 0 & F \end{array}\right]\leq \mathrm{rank}\left[\begin{array}{cc} sI_{n}-A & B\\ \tilde{C} & 0 \end{array}\right]+r \end{array}$$

which gives $n+m\leq \mathrm{rank}\left[\begin{array}{cc} sI_{n}-A & B\\ \tilde{C} & 0 \end{array}\right]\leq n+m$. That is, $\mathrm{rank}\left[\begin{array}{cc} sI_{n}-A & B\\ \tilde{C} & 0 \end{array}\right]=n+m$ is true for all complex *s* with Re(s)≥0. Moreover, as

$$\begin{array}{cc} \mathrm{rank}\left[\begin{array}{cc} sI_{n}-A & B\\ \overset{⌢}{C} & 0 \end{array}\right] & =\mathrm{rank}\left[\begin{array}{cc} sI_{n}-A & B\\ \Upsilon \tilde{C} & 0 \end{array}\right]\\ \; & =\mathrm{rank}\left\{\left[\begin{array}{cc} I_{n} & \\ \; & \Upsilon \end{array}\right]\left[\begin{array}{cc} sI_{n}-A & B\\ \tilde{C} & 0 \end{array}\right]\right\}\\ \; & =\mathrm{rank}\left[\begin{array}{cc} sI_{n}-A & B\\ \tilde{C} & 0 \end{array}\right]=n+m \end{array}$$

then we can conclude that (7) holds for all complex *s* with Re(s)≥0. □

**Lemma** **5.** *Under Assumption 2, we have $\mathrm{rank}\left(\overset{⌢}{C}B\right)=\mathrm{rank}\left(B\right)$.*

**Proof.**  It follows from (4) in Assumption 2 that

$$\begin{array}{cc} n+m+r & =\mathrm{rank}\left[\begin{array}{ccc} I_{n} & B & 0\\ C & 0 & F \end{array}\right]\\ \; & =\mathrm{rank}3\{\left[\begin{array}{cc} I_{n} & 0\\ 0 & FF^{†}\\ 0 & I_{p}-FF^{†} \end{array}\right]\left[\begin{array}{ccc} I_{n} & B & 0\\ C & 0 & F \end{array}\right]\left[\begin{array}{ccc} I_{n} & 0 & 0\\ 0 & I_{m} & 0\\ -F^{†}C & 0 & I_{r} \end{array}\right]3\}\\ \; & =\mathrm{rank}\left\{\left[\begin{array}{cc} I_{n} & 0\\ 0 & FF^{†}\\ 0 & I_{p}-FF^{†} \end{array}\right]\left[\begin{array}{ccc} I_{n} & B & 0\\ \tilde{C} & 0 & F \end{array}\right]\right\}\\ \; & =\mathrm{rank}\left[\begin{array}{ccc} I_{n} & B & 0\\ FF^{†}\tilde{C} & 0 & I_{r}\\ \left(I_{p}-FF^{†}\right)\tilde{C} & 0 & 0 \end{array}\right]\\ \; & =\mathrm{rank}\left[\begin{array}{ccc} I_{n} & B & 0\\ 0 & 0 & I_{r}\\ \left(I_{p}-FF^{†}\right)\tilde{C} & 0 & 0 \end{array}\right]\\ \; & =\mathrm{rank}\left[\begin{array}{cc} I_{n} & B\\ \left(I_{p}-FF^{†}\right)\tilde{C} & 0 \end{array}\right]+r\\ \; & =\mathrm{rank}\left\{\left[\begin{array}{cc} I_{n} & \\ & I_{p}-FF^{†} \end{array}\right]\left[\begin{array}{cc} I_{n} & B\\ \tilde{C} & 0 \end{array}\right]\right\}+r\\ \; & \leq \mathrm{rank}\left[\begin{array}{cc} I_{n} & B\\ \tilde{C} & 0 \end{array}\right]+r\\ \; & =\mathrm{rank}\left\{\left[\begin{array}{cc} I_{n} & \\ \; & \Upsilon \end{array}\right]\left[\begin{array}{cc} I_{n} & B\\ \tilde{C} & 0 \end{array}\right]\right\}+r\\ \; & =\mathrm{rank}\left[\begin{array}{cc} I_{n} & B\\ \left[\begin{array}{c} \overset{⌢}{C}\\ 0_{r\times n} \end{array}\right] & \left[\begin{array}{c} 0_{\overset{⌢}{p}\times m}\\ 0_{r\times m} \end{array}\right] \end{array}\right]+r\\ \; & =\mathrm{rank}\left[\begin{array}{cc} I_{n} & B\\ \overset{⌢}{C} & 0_{\overset{⌢}{p}\times m} \end{array}\right]+r \end{array}$$

Thus, $n+m\leq \mathrm{rank}\left[\begin{array}{cc} I_{n} & B\\ \overset{⌢}{C} & 0 \end{array}\right]\leq n+m$ which is equivalent to $\mathrm{rank}\left[\begin{array}{cc} I_{n} & B\\ \overset{⌢}{C} & 0 \end{array}\right]=n+m$. Furthermore, we have

$$\begin{array}{cc} n+m & =\mathrm{rank}\left[\begin{array}{cc} I_{n} & B\\ \overset{⌢}{C} & 0 \end{array}\right]\\ \; & =\mathrm{rank}\left\{\left[\begin{array}{cc} I_{n} & 0\\ -\overset{⌢}{C} & I_{p} \end{array}\right]\left[\begin{array}{cc} I_{n} & B\\ \overset{⌢}{C} & 0 \end{array}\right]\left[\begin{array}{cc} I_{n} & B\\ 0 & I_{m} \end{array}\right]\right\}\\ \; & =\mathrm{rank}\left[\begin{array}{cc} I_{n} & 0\\ 0 & -\overset{⌢}{C}B \end{array}\right]=n+\mathrm{rank}(\overset{⌢}{C}B) \end{array}$$

which means that $\mathrm{rank}\left(\overset{⌢}{C}B\right)=m=\mathrm{rank}\left(B\right)$. □

### 3.2. Reduced-Order Observer Design

A reduced-order observer is designed for (6) to obtain the state estimation and SFR in this subsection.

**Lemma** **6** ([42]). *Under Assumptions 1 and 2, for some positive symmetric definite matrix $Q\in R^{n\times n}$, there exist $K\in R^{n\times \overset{⌢}{p}}$ and $H\in R^{q\times \overset{⌢}{p}}$, and a positive symmetric definite matrix $P\in R^{n\times n}$ such that*

(8) $$\begin{array}{c} \left\{\begin{array}{cc} \; & {\left(A-K\overset{⌢}{C}\right)}^{T}P+P\left(A-K\overset{⌢}{C}\right)=-Q\\ \; & B^{T}P=H\overset{⌢}{C} \end{array}\right. \end{array}$$

*holds.*

*Based on Smith orthogonal procedure, there exists a nonsingular matrix $S\in R^{\overset{⌢}{p}\times \overset{⌢}{p}}$ satisfying $\overset{⌢}{C}=S\overset{˘}{C}$, where $\overset{˘}{C}\in R^{\overset{⌢}{p}\times n}$ and $\overset{˘}{C}{\overset{˘}{C}}^{T}=I_{\overset{⌢}{p}}$. Next, we extend $\overset{˘}{C}$ to be an n×n orthogonal matrix $W={\left[\begin{array}{cc} {\overset{˘}{C}}^{T} & M^{T} \end{array}\right]}^{T}$, where $M\in R^{(n-\overset{⌢}{p})\times n}$. Now, we make an equivalent state transformation z=Wx, and the system (6) or (1) becomes*

(9) $$\begin{array}{c} \left\{\begin{array}{cc} \dot{z} & =\bar{A}z+\bar{B}\left(u+w\right)\\ \overset{⌢}{y} & =\bar{C}z \end{array}\right. \end{array}$$

*where $\bar{A}=WAW^{T},\bar{B}=WB$, and $\bar{C}=\overset{⌢}{C}W^{T}=\left[\begin{array}{cc} S & 0 \end{array}\right]\in R^{\overset{⌢}{p}\times n}$.*

**Lemma** **7.** *For $\bar{Q}=WQW^{T},\bar{K}=WK,\bar{P}=WPW^{T}$, and $\bar{H}=H$, we have*

(10) $$\begin{array}{c} \left\{\begin{array}{cc} \; & {\left(\bar{A}-\bar{K}\overset{⌢}{C}\right)}^{T}\bar{P}+\bar{P}\left(\bar{A}-\bar{K}\bar{C}\right)=-\bar{Q}\\ \; & {\bar{B}}^{T}\bar{P}=\bar{H}\bar{C} \end{array}\right. \end{array}$$

**Proof.**  The proof of Lemma 7 can be completed by verifying it directly.

In the following, we make the block vector or matrix decomposition: $z=\left[\begin{array}{c} z_{1}\\ z_{2} \end{array}\right],\bar{B}=\left[\begin{array}{c} {\bar{B}}_{1}\\ {\bar{B}}_{2} \end{array}\right]$, $\bar{K}=\left[\begin{array}{c} {\bar{K}}_{1}\\ {\bar{K}}_{2} \end{array}\right],\bar{A}=\left[\begin{array}{cc} {\bar{A}}_{11} & {\bar{A}}_{12}\\ {\bar{A}}_{21} & {\bar{A}}_{22} \end{array}\right],\bar{P}=\left[\begin{array}{cc} {\bar{P}}_{1} & {\bar{P}}_{2}\\ {\bar{P}}_{2}^{T} & {\bar{P}}_{3} \end{array}\right]$ and $\bar{Q}=\left[\begin{array}{cc} {\bar{Q}}_{1} & {\bar{Q}}_{2}\\ {\bar{Q}}_{2}^{T} & {\bar{Q}}_{3} \end{array}\right]$, where $z_{1}\in R^{\overset{⌢}{p}}$, ${\bar{B}}_{1}\in R^{\overset{⌢}{p}\times m},{\bar{K}}_{1}\in R^{\overset{⌢}{p}\times p},{\bar{A}}_{11}\in R^{\overset{⌢}{p}\times \overset{⌢}{p}},{\bar{P}}_{1}\in R^{\overset{⌢}{p}\times \overset{⌢}{p}}$ and ${\bar{Q}}_{1}\in R^{\overset{⌢}{p}\times \overset{⌢}{p}}$. By the second equation of (10), and recalling that $\bar{C}=\left[\begin{array}{cc} S & 0 \end{array}\right]$, we can deduce that $\bar{P}\bar{B}=\left[\begin{array}{c} S^{T}\bar{H}\\ 0 \end{array}\right]\Rightarrow \left[\begin{array}{cc} 0 & I_{n-\overset{⌢}{p}} \end{array}\right]\bar{P}\bar{B}=0\Leftrightarrow \left[\begin{array}{cc} {\bar{P}}_{2}^{T} & {\bar{P}}_{3} \end{array}\right]\bar{B}=0\Leftrightarrow \left[\begin{array}{cc} {\bar{P}}_{3}^{-1}{\bar{P}}_{2}^{T} & I_{n-\overset{⌢}{p}} \end{array}\right]\bar{B}=0$. That is, we have $\left[\begin{array}{cc} \bar{L} & I_{n-\overset{⌢}{p}} \end{array}\right]\bar{B}=0$, where $\bar{L}={\bar{P}}_{3}^{-1}{\bar{P}}_{2}^{T}$. Making a further state equivalent transformation of $\zeta =\left[\begin{array}{c} \zeta_{1}\\ \zeta_{2} \end{array}\right]=\left[\begin{array}{cc} I_{\overset{⌢}{p}} & 0\\ \bar{L} & I_{n-\overset{⌢}{p}} \end{array}\right]\left[\begin{array}{c} z_{1}\\ z_{2} \end{array}\right]$ for (9) and using $\left[\begin{array}{cc} \bar{L} & I_{n-p} \end{array}\right]\bar{B}=0$ gives

(11) $$\begin{array}{c} \left\{\begin{array}{cc} {\dot{\zeta }}_{1} & =\left({\bar{A}}_{11}-{\bar{A}}_{12}\bar{L}\right)\zeta_{1}+{\bar{A}}_{12}\zeta_{2}+{\bar{B}}_{1}\left(u+w\right)\\ {\dot{\zeta }}_{2} & =\left({\bar{A}}_{22}+\bar{L}{\bar{A}}_{12}\right)\zeta_{2}+{\overset{˘}{A}}_{2}\zeta_{1}\\ \overset{⌢}{y} & =S\zeta_{1} \end{array}\right. \end{array}$$

where ${\overset{˘}{A}}_{2}=\bar{L}{\bar{A}}_{11}+{\bar{A}}_{21}-\left(\bar{L}{\bar{A}}_{12}+{\bar{A}}_{22}\right)\bar{L}$. Now, for the equivalent system (11), we design a reduced-order observer as follows:

(12) $$\begin{array}{c} \left\{\begin{array}{cc} \dot{\hat{\zeta_{2}}} & =\left({\bar{A}}_{22}+\bar{L}{\bar{A}}_{12}\right){\hat{\zeta }}_{2}+{\overset{˘}{A}}_{2}S^{-1}\overset{⌢}{y}\\ \hat{x} & =W^{T}\left[\begin{array}{c} S^{-1}\overset{⌢}{y}\\ {\hat{\zeta }}_{2}-\bar{L}S^{-1}\overset{⌢}{y} \end{array}\right] \end{array}\right. \end{array}$$

□

**Theorem** **1.** *Under Assumptions 1 and 2, (12) is a reduced-order observer with dimension $n-\overset{⌢}{p}$ of (1) or (9), such that $\lim_{t\to \infty }{\tilde{\zeta }}_{2}\left(t\right)=0$ and, thus, $\lim_{t\to \infty }\tilde{x}\left(t\right)=0$, even if the original system (1) suffers from actuator and sensor faults simultaneously, where ${\tilde{\zeta }}_{2}=\zeta_{2}-{\hat{\zeta }}_{2}$ and $\tilde{x}=x-\hat{x}$.*

**Proof.**  On one hand, the observer error dynamic system can be obtained from the first equation of (12) and the second equation of (11):

(13) $$\begin{array}{c} {\dot{\tilde{\zeta }}}_{2}=\left({\bar{A}}_{22}+\bar{L}{\bar{A}}_{12}\right){\tilde{\zeta }}_{2} \end{array}$$

On the other hand, by the first equation of (10), we have

$$\begin{array}{c} \left[\begin{array}{cc} He\left({\bar{P}}_{1}\left({\bar{A}}_{11}-{\bar{K}}_{1}S\right)+{\bar{P}}_{2}\left({\bar{A}}_{21}-{\bar{K}}_{2}S\right)\right) & He\left({\bar{P}}_{1}{\bar{A}}_{12}+{\bar{P}}_{2}{\bar{A}}_{22}\right)\\ {}* & {\bar{P}}_{3}\left({\bar{A}}_{22}+\bar{L}{\bar{A}}_{12}\right)+{\left({\bar{A}}_{22}+\bar{L}{\bar{A}}_{12}\right)}^{T}{\bar{P}}_{3} \end{array}\right]\\ =-\left[\begin{array}{cc} {\bar{Q}}_{1} & {\bar{Q}}_{2}\\ {\bar{Q}}_{2}^{T} & {\bar{Q}}_{3} \end{array}\right] \end{array}$$

which contains

(14) $$\begin{array}{c} {\bar{P}}_{3}\left({\bar{A}}_{22}+\bar{L}{\bar{A}}_{12}\right)+{\left({\bar{A}}_{22}+\bar{L}{\bar{A}}_{12}\right)}^{T}{\bar{P}}_{3}=-{\bar{Q}}_{3}<0 \end{array}$$

By Lyapunov stability, we known that (14) implies that dynamic system (13) is asymptotically stable. Consequently, we can conclude that $\lim_{t\to \infty }{\tilde{\zeta }}_{2}\left(t\right)=0$ and, thus, $\lim_{t\to \infty }\tilde{x}\left(t\right)=W^{T}\left[\begin{array}{c} 0\\ \lim_{t\to \infty }{\tilde{\zeta }}_{2}\left(t\right) \end{array}\right]=0$. Moreover, from the output equation in (1), a sensor fault reconstruction method is developed based on the reduced-order observer (12) as ${\hat{f}}_{s}=F^{†}\left(y-C\hat{x}\right)$. □

## 4. The Designs of MUIR and FTC

In this section, we firstly develop an MUIR method via an interval observer, and then an observer-based FTC scheme is developed.

### 4.1. MUIR Design

It follows from (11) that

(15) $$\begin{array}{c} \dot{\overset{⌢}{y}}=S\left({\bar{A}}_{11}-{\bar{A}}_{12}\bar{L}\right)S^{-1}\overset{⌢}{y}+S{\bar{A}}_{12}\zeta_{2}+S{\bar{B}}_{1}u+S{\bar{B}}_{1}w \end{array}$$

For (15), we design an interval observer to obtain the interval estimation of $\overset{⌢}{y}$ as follows:

(16) $$\begin{array}{c} \left\{\begin{array}{cc} \dot{\bar{\overset{⌢}{y}}} & =S\left({\bar{A}}_{11}-{\bar{A}}_{12}\bar{L}\right)S^{-1}\overset{⌢}{y}+S{\bar{A}}_{12}{\hat{\zeta }}_{2}+S{\bar{B}}_{1}u\\ \; & +{\left(S{\bar{B}}_{1}\right)}^{+}\bar{w}-{\left(S{\bar{B}}_{1}\right)}^{-}\underline{w}-\Lambda \left(\overset{⌢}{y}-\bar{\overset{⌢}{y}}\right)\\ \dot{\underline{\overset{⌢}{y}}} & =S\left({\bar{A}}_{11}-{\bar{A}}_{12}\bar{L}\right)S^{-1}\overset{⌢}{y}+S{\bar{A}}_{12}{\hat{\zeta }}_{2}+S{\bar{B}}_{1}u\\ \; & +{\left(S{\bar{B}}_{1}\right)}^{+}\underline{w}-{\left(S{\bar{B}}_{1}\right)}^{-}\bar{w}-\Lambda \left(\overset{⌢}{y}-\underline{\overset{⌢}{y}}\right) \end{array}\right. \end{array}$$

where ${\hat{\zeta }}_{2}$ is provided by reduced-order observer (12).

**Theorem** **2.** *Under Assumptions 1 and 3, (16) is an interval observer of (15), which can lead to $\underline{\overset{⌢}{y}}\left(t\right)\leq \overset{⌢}{y}\left(t\right)\leq \bar{\overset{⌢}{y}}\left(t\right)$ holding for all $t\geq c_{0}$ for some positive scalar $c_{0}>0$, if* Λ *is selected to be a Metzler and Hurwitz matrix arbitrarily, and the initial values are determined as $\bar{\overset{⌢}{y}}\left(0\right)={\overset{⌢}{C}}^{+}{\bar{x}}_{0}-{\overset{⌢}{C}}^{-}{\underline{x}}_{0}$ and $\underline{\overset{⌢}{y}}\left(0\right)={\overset{⌢}{C}}^{+}{\underline{x}}_{0}-{\overset{⌢}{C}}^{-}{\bar{x}}_{0}$.*

**Proof.**  The interval observer error system can be obtained through (15) and (16):

(17) $$\begin{array}{c} \left\{\begin{array}{c} {\dot{\bar{e}}}_{\overset{⌢}{y}}=\Lambda {\bar{e}}_{\overset{⌢}{y}}-S{\bar{A}}_{12}{\tilde{\zeta }}_{2}+{\left(S{\bar{B}}_{1}\right)}^{+}\bar{w}-{\left(S{\bar{B}}_{1}\right)}^{-}\underline{w}-S{\bar{B}}_{1}w\\ {\dot{\underline{e}}}_{\overset{⌢}{y}}=\Lambda {\underline{e}}_{\overset{⌢}{y}}+S{\bar{A}}_{12}{\tilde{\zeta }}_{2}+{\left(S{\bar{B}}_{1}\right)}^{-}\bar{w}-{\left(S{\bar{B}}_{1}\right)}^{+}\underline{w}+S{\bar{B}}_{1}w \end{array}\right. \end{array}$$

where ${\overset{⌢}{e}}_{\overset{⌢}{y}}=\bar{\overset{⌢}{y}}-\overset{⌢}{y}$ and ${\underline{e}}_{\overset{⌢}{y}}=\overset{⌢}{y}-\underline{\overset{⌢}{y}}$. By Lemma 1, ${\left(S{\bar{B}}_{1}\right)}^{+}\bar{w}-{\left(S{\bar{B}}_{1}\right)}^{-}\underline{w}-S{\bar{B}}_{1}w>0$ and ${\left(S{\bar{B}}_{1}\right)}^{-}\bar{w}-{\left(S{\bar{B}}_{1}\right)}^{+}\underline{w}+S{\bar{B}}_{1}w>0$ hold. Thus, there exists $c_{0}>0$ such that $-S{\bar{A}}_{12}{\tilde{\zeta }}_{2}+{\left(S{\bar{B}}_{1}\right)}^{+}\bar{w}-{\left(S{\bar{B}}_{1}\right)}^{-}\underline{w}-S{\bar{B}}_{1}w$ and $S{\bar{A}}_{12}{\tilde{\zeta }}_{2}+{\left(S{\bar{B}}_{1}\right)}^{-}\bar{w}-{\left(S{\bar{B}}_{1}\right)}^{+}\underline{w}+S{\bar{B}}_{1}w\geq 0$ hold for all $t\geq c_{0}$. In addition, Assumption 3 indicates that ${\underline{x}}_{0}\leq x\left(0\right)\leq {\bar{x}}_{0}$, By Lemma 1, again, one has $\underline{\overset{⌢}{y}}\left(0\right)\leq \overset{⌢}{y}\left(0\right)\leq \bar{\overset{⌢}{y}}\left(0\right)$, which is equivalent to ${\bar{e}}_{\overset{⌢}{y}}\left(0\right)\geq 0$ and ${\underline{e}}_{\overset{⌢}{y}}\left(0\right)\geq 0$. Now, if the Λ is Hurwitz and Metzler, applying Lemma 2 on (17) yields ${\bar{e}}_{\overset{⌢}{y}}\left(t\right)\geq 0$ and ${\underline{e}}_{\overset{⌢}{y}}\left(t\right)\geq 0$, which imply that $\underline{\overset{⌢}{y}}\left(t\right)\leq \overset{⌢}{y}\left(t\right)\leq \bar{\overset{⌢}{y}}\left(t\right)$ holds for all $t\geq c_{0}$.

Based on the output upper and lower bound estimates, we aim to establish an algebraic connection of the MUI and the state estimation error. Initially, as $\underline{\overset{⌢}{y}}\leq \overset{⌢}{y}\leq \bar{\overset{⌢}{y}}$, there exists a time-varying vector α(t) such that

(18) $$\begin{array}{c} \overset{⌢}{y}=\mathrm{diag}\left(\overset{˘}{y}\right)\alpha +\underline{\overset{⌢}{y}} \end{array}$$

where $\alpha =\left[\begin{array}{ccc} \alpha_{1} & \cdots & \alpha_{\overset{⌢}{p}} \end{array}\right]$ with $0\leq \alpha_{i}\leq 1\left(i=1,\cdots ,\overset{⌢}{p}\right)$ and $\overset{˘}{y}=\bar{\overset{⌢}{y}}-\underline{\overset{⌢}{y}}$. Then, one has

(19) $$\begin{array}{c} \alpha =\left({\mathrm{diag}}^{-1}\left(\overset{˘}{y}+\varepsilon \right)-\mathrm{diag}\left(\varepsilon \right)\right)\left(\overset{⌢}{y}-\underline{\overset{⌢}{y}}\right) \end{array}$$

where $\varepsilon =\left[\begin{array}{ccc} \varepsilon_{1} & \cdots & \varepsilon_{\overset{⌢}{p}} \end{array}\right]$ is a time-varying vector with $\varepsilon_{i}=1$ if ${\overset{˘}{y}}_{i}=0$, otherwise $\varepsilon_{i}=0\left(i=1,\ldots ,\overset{⌢}{p}\right)$. Meanwhile, by (18), we have

(20) $$\begin{array}{c} \dot{\overset{⌢}{y}}=\mathrm{diag}\left(\dot{\overset{˘}{y}}\right)\alpha +\mathrm{diag}\left(\overset{˘}{y}\right)\dot{\alpha }+\dot{\overset{⌢}{\underline{y}}} \end{array}$$

It comes from (16) that

(21) $$\begin{array}{c} \dot{\overset{˘}{y}}=\phi_{1}=\Lambda \overset{˘}{y}+\left|S{\bar{B}}_{1}\right|\bar{w}-\left|S{\bar{B}}_{1}\right|\underline{w} \end{array}$$

where $\overset{˘}{w}=\bar{w}-\underline{w}$. Consequently, substituting the second equation of (16) and (21) into (20) gives

(22) $$\begin{array}{c} \dot{\overset{⌢}{y}}=\mathrm{diag}\left(\phi_{1}\right)\alpha +\mathrm{diag}\left(\overset{˘}{y}\right)\dot{\alpha }+\phi_{2}+S{\bar{A}}_{12}{\hat{\zeta }}_{2}+S{\bar{B}}_{1}u \end{array}$$

where $\phi_{2}=S\left({\bar{A}}_{11}-{\bar{A}}_{12}\bar{L}\right)S^{-1}\overset{⌢}{y}+{\left(S{\bar{B}}_{1}\right)}^{+}\underline{w}-{\left(S{\bar{B}}_{1}\right)}^{-}\bar{w}-\Lambda \left(\overset{⌢}{y}-\underline{\overset{⌢}{y}}\right)$. Comparing (15) with (22), we obtain

(23) $$S{\bar{B}}_{1}w=\mathrm{diag}\left(\phi_{1}\right)\alpha +\mathrm{diag}\left(\overset{˘}{y}\right)\dot{\alpha }+\phi_{2}-S\left({\bar{A}}_{11}-{\bar{A}}_{12}\bar{L}\right)S^{-1}S\overset{⌢}{y}-S{\bar{A}}_{12}{\tilde{\zeta }}_{2}$$

Note that $\overset{⌢}{C}B=\bar{C}\bar{B}=S\left[\begin{array}{cc} I_{\overset{⌢}{p}} & 0 \end{array}\right]\;\bar{B}=S{\bar{B}}_{1}$; thus, from (23), we have

(24) $$w={\left(\overset{⌢}{C}B\right)}^{†}\left[\mathrm{diag}\left(\phi_{1}\right)\alpha +\mathrm{diag}\left(\overset{˘}{y}\right)\dot{\alpha }+\phi_{2}-S\left({\bar{A}}_{11}-{\bar{A}}_{12}\bar{L}\right)S^{-1}\overset{⌢}{y}-S{\bar{A}}_{12}{\tilde{\zeta }}_{2}\right]$$

where $\left(\overset{⌢}{C}B\right)={\left[{\left(\overset{⌢}{C}B\right)}^{T}\left(\overset{⌢}{C}B\right)\right]}^{-1}{\left(\overset{⌢}{C}B\right)}^{T}$, and its existence can be guaranteed by Lemma 5. Now, by referring to (24), an MUIR method is developed by setting ${\tilde{\zeta }}_{2}=0$

(25) $$\hat{w}={\left(\bar{C}\bar{B}\right)}^{†}\left[\mathrm{diag}\left(\phi_{1}\right)\alpha +\mathrm{diag}\left(\overset{˘}{y}\right)\hat{\dot{\alpha }}+\phi_{2}-S\left({\bar{A}}_{11}-{\bar{A}}_{12}\bar{L}\right)S^{-1}\overset{⌢}{y}\right]$$

where α is determined by (19), and ${\hat{\dot{\alpha }}}_{i}$ is taken as the identical estimation of ${\dot{\alpha }}_{i}$ in a finite time generated by the following differentiator [43]:

(26) $$\begin{array}{c} \left\{\begin{array}{cc} {\dot{\xi }}_{1,i} & =\kappa_{1,i},\\ \kappa_{1,i} & =-\gamma_{1,i}{\left|\xi_{1,i}-\alpha_{i}\right|}^{\frac{1}{2}}\mathrm{sign}\left(\xi_{1,i}-\alpha_{i}\right)+\xi_{2,i}\\ {\dot{\xi }}_{2,i} & =-\gamma_{2,i}\mathrm{sign}\left(\xi_{2,i}-\kappa_{1,i}\right) \end{array},\right. \end{array}$$

where $\gamma_{1,i},\gamma_{2,i}>0\left(i=1,\cdots ,p\right)$ are two positive scalar gains that need to be selected properly. Then $\xi_{2,i}$ serves as the identical estimation of ${\dot{\alpha }}_{i}$ in a finite time, and, thus, ${\hat{\dot{\alpha }}}_{i}={\left[\begin{array}{ccc} \xi_{2,1} & \cdots & \xi_{2,\overset{⌢}{p}} \end{array}\right]}^{T}$ is the identical estimation of ${\dot{\alpha }}_{i}$ in a finite time. Subtracting (25) from (24) yields

(27) $$\begin{array}{c} \tilde{w}=-{\left(\bar{C}\bar{B}\right)}^{†}S{\bar{A}}_{12}{\tilde{\zeta }}_{2} \end{array}$$

where $\tilde{w}=w-\hat{w}$. Obviously, we have $\lim_{t\to \infty }\tilde{w}\left(t\right)=-{\left(\bar{C}\bar{B}\right)}^{†}S{\bar{A}}_{12}\lim_{t\to \infty }{\tilde{\zeta }}_{2}\left(t\right)=0$. □

**Remark** **2.** *MUIR (25) totally depends on the interval observer (16) and the differentiator (26). It is an algebraic one and is able to asymptotically estimate the actual MUI. Moreover, it should be emphasized that MUIR (25) decouples the control input u(t). It is through this significant feature that the following controller (28) can realize the challenging task highlighted in Remark 1.*

### 4.2. FTC Design

In this subsection, an FTC scheme is proposed based on the reduced-order observer (12) together with MUIR (25), which is given through the interval observer (16).

For System (1) and based on (5), we design FTC:

(28) $$\begin{array}{c} u=U\hat{x}-\hat{w} \end{array}$$

where $U\in R^{m\times n}$ is the feedback gain matrix, $\hat{x}$ is given by the reduced-order observer (12), and $\hat{w}$ is determined by (25). Now, substituting (28) into the first equation of (5) leads to

(29) $$\begin{array}{c} \dot{x}=\left(A+BU\right)x-BU\tilde{x}+B\tilde{w} \end{array}$$

Furthermore, substituting (27) into (29) gives

(30) $$\begin{array}{c} \dot{x}=\left(A+BU\right)x-BU\tilde{x}-B{\left(\bar{C}\bar{B}\right)}^{†}S{\bar{A}}_{12}{\tilde{\zeta }}_{2} \end{array}$$

Note that $\tilde{x}=W^{T}\left[\begin{array}{c} 0\\ {\tilde{\zeta }}_{2} \end{array}\right]=\left[\begin{array}{cc} {\overset{˘}{C}}^{T} & M^{T} \end{array}\right]\left[\begin{array}{c} 0\\ {\tilde{\zeta }}_{2} \end{array}\right]=M^{T}{\tilde{\zeta }}_{2}$, and (30) becomes

(31) $$\begin{array}{c} \dot{x}=\left(A+BU\right)x-B\left(UM^{T}+{\left(\bar{C}\bar{B}\right)}^{†}S{\bar{A}}_{12}\right){\tilde{\zeta }}_{2} \end{array}$$

From (31) and (13), the observer-based control closed-loop system is

(32) $$\begin{array}{c} \left[\begin{array}{c} \dot{x}\\ {\dot{\tilde{\zeta }}}_{2} \end{array}\right]=\left[\begin{array}{cc} A+BU & -B\left(UM^{T}+{\left(\bar{C}\bar{B}\right)}^{†}S{\bar{A}}_{12}\right)\\ 0 & {\bar{A}}_{22}+\bar{L}{\bar{A}}_{12} \end{array}\right]\left[\begin{array}{c} x\\ {\tilde{\zeta }}_{2} \end{array}\right] \end{array}$$

**Remark** **3.** *The reduced-order-observer-based state and MUIR feedback controller (28) form the FTC, and (32) indicates that the FTC through the state feedback together with an MUIR has no relation to the eigenvalues of the original state feedback. The eigenvalues of the reduced-order observer (12) are not affected by the connection either. For this reason, the design of the state feedback gain matrix U, together with the design of the MUIR (25), and the reduced-order observer (12) can be fulfilled independently, and then just connected. This is called the separation property.*

## 5. Simulations

This section presents two simulation examples containing a numerical simulation and a simulation for a practical system to demonstrate the effectiveness of the proposed approach.

### 5.1. Example 1

Consider an uncertain system (1) with system matrices as

$$\begin{array}{cc} A & =\left[\begin{array}{cccc} 5 & -3 & 1 & 1\\ 3 & -4 & 6 & 0\\ 0 & -3 & -5 & 0\\ 1 & -3 & 0 & -1 \end{array}\right],\;\;\;\;B=\left[\begin{array}{c} 0\\ 1\\ 0\\ 0 \end{array}\right],\;\;\;\;C=\left[\begin{array}{cccc} 1 & 0 & 0 & 0\\ 0 & 1 & 0 & 0\\ 0 & 0 & 0 & 1 \end{array}\right],\;\;\;\;F=\left[\begin{array}{c} 1\\ 0\\ 0 \end{array}\right]. \end{array}$$

Thus, one has n=4,m=1 and r=1. For Assumption 1, by calling the MATLAB R2024a function of z = tzero(A, [ B zeros(4, 1)], C, [zeros(3, 1) F]), it returns an empty vector to z, which means that the system has no invariant zero. Consequently, Assumption 1 is satisfied. For Assumption 2, we can verify it directly by calling the MATLAB function of g = rank([eye(4) B zeros(4, 1); C zeros(3, 1) F]), and one obtains that g = 6 = n + m + r. Therefore, Assumption 2 is satisfied, and we point out that the system is unstable because it contains an eigenvalue of 4.002. In addition, we assume that the external disturbance is d=sin(2t−3). In order to demonstrate the performances of the fault reconstruction and FTC, we assume that

$$\begin{array}{cc} \rho & =\left\{\begin{array}{cc} \; & 0,t\leq \;5\\ \; & 0.2,t>5 \end{array},\right.\;\;\;\;f_{a}=\left\{\begin{array}{cc} \; & 1,t\leq \;5\\ \; & 1.5\sin(3t),t>5 \end{array},\right.\;\;\;\;f_{s}=\left\{\begin{array}{cc} \; & 0,t\leq \;5\\ \; & 0.5\cos(1.5t-1)+1,t>5 \end{array}.\right. \end{array}$$

Firstly, it is not difficult for us to obtain $\tilde{C}=\left[\begin{array}{cccc} 0 & 0 & 0 & 0\\ 0 & 1 & 0 & 0\\ 0 & 0 & 0 & 1 \end{array}\right]$ by calculation. Secondly, by selecting $\Upsilon =\left[\begin{array}{ccc} 0 & 1 & 0\\ 0 & 0 & 1\\ 1 & 0 & 0 \end{array}\right]$, the matrix $\overset{⌢}{C}$ can be obtained as $\overset{⌢}{C}=\left[\begin{array}{cccc} 0 & 1 & 0 & 0\\ 0 & 0 & 0 & 1 \end{array}\right]$ which is a matrix having full row rank. Thirdly, by using Smith orthogonal procedure, there exists a nonsingular matrix $S=\left[\begin{array}{cc} 1 & 0\\ 0 & 1 \end{array}\right]$ and $\overset{˘}{C}=\overset{⌢}{C}$ meeting $\overset{⌢}{C}=S\overset{˘}{C}$, where $\overset{˘}{C}\in R^{\overset{⌢}{p}\times n}$ and $\overset{˘}{C}{\overset{˘}{C}}^{T}=I_{\overset{⌢}{p}}$. Next, we extend $\overset{˘}{C}$ to be an n×n orthogonal matrix:

$$\begin{array}{c} W=\left[\begin{array}{cccc} 0 & 1 & 0 & 0\\ 0 & 0 & 0 & 1\\ 0 & 0 & 1 & 0\\ 1 & 0 & 0 & 0 \end{array}\right] \end{array}$$

Moreover, solving (8) gives

$$\begin{array}{cc} P & =10^{6}\times \left[\begin{array}{cccc} 0.0465 & 0.0000 & 0.0052 & -0.3049\\ 0.0000 & 0.0002 & 0.0000 & 0.0146\\ 0.0052 & 0.0000 & 0.2000 & -0.0396\\ -0.3049 & 0.0146 & -0.0396 & 3.0919 \end{array}\right],\\ K & =10^{5}\times \left[\begin{array}{cc} -0.0748 & 0.6566\\ 0.8567 & -7.4845\\ -0.0003 & 0.0026\\ -0.0114 & 0.1001 \end{array}\right],H=10^{4}\times \left[\begin{array}{cc} 0.0200 & 1.4573 \end{array}\right]. \end{array}$$

Thus, we have

$$\begin{array}{cc} \bar{A} & =\left[\begin{array}{cccc} -4 & 0 & 6 & 3\\ -3 & -1 & 0 & 1\\ -3 & 0 & -5 & 0\\ -3 & 1 & 1 & 5 \end{array}\right],\bar{B}=\left[\begin{array}{c} 1\\ 0\\ 0\\ 0 \end{array}\right],\bar{C}=\left[\begin{array}{cccc} 1 & 0 & 0 & 0\\ 0 & 1 & 0 & 0 \end{array}\right],\\ \bar{P} & =10^{6}\times \left[\begin{array}{cccc} 0.0002 & 0.0146 & 0.0000 & 0.0000\\ 0.0146 & 3.0919 & -0.0396 & -0.3049\\ 0.0000 & -0.0396 & 0.2000 & 0.0052\\ 0.0000 & -0.3049 & 0.0052 & 0.0465 \end{array}\right],\\ \bar{K} & =10^{5}\times \left[\begin{array}{cc} 0.8567 & -7.4845\\ -0.0114 & 0.1001\\ -0.0003 & 0.0026\\ -0.0748 & 0.6566 \end{array}\right], \end{array}$$

which gives ${\bar{A}}_{11}=\left[\begin{array}{cc} -4 & 0\\ -3 & -1 \end{array}\right]$, ${\bar{A}}_{21}=\left[\begin{array}{cc} -3 & 0\\ -3 & 1 \end{array}\right]$, ${\bar{A}}_{12}=\left[\begin{array}{cc} 6 & 3\\ 0 & 1 \end{array}\right]$, ${\bar{A}}_{22}=\left[\begin{array}{cc} -5 & 0\\ 1 & 5 \end{array}\right]$, ${\bar{P}}_{2}=10^{6}\times \left[\begin{array}{cc} 0.0000 & -0.0396\\ 0.0000 & -0.3049 \end{array}\right]$ and ${\bar{P}}_{3}=10^{6}\times \left[\begin{array}{cc} 0.2000 & 0.0052\\ 0.0052 & 0.0465 \end{array}\right]$. As a result, we can calculate $\bar{L}=\left[\begin{array}{cc} -0.0000 & -0.0261\\ 0.0000 & -6.5562 \end{array}\right],{\overset{˘}{A}}_{2}=\left[\begin{array}{cc} -2.9218 & -0.2753\\ 16.6686 & -2.6206 \end{array}\right]$. Finally, based on these matrix parameters, the reduced-order observer (12) can be constructed.

Next, in order to construct the interval observer (16), the initial values and some parameters are given as $\underline{u}=-15$, $\bar{u}=15,\underline{d}=-1,\bar{d}=1,{\underline{x}}_{0}=x\left(0\right)-0.31_{4},{\bar{x}}_{0}=x\left(0\right)+0.31_{4},\underline{\rho }=0.01,\bar{\rho }=1$, ${\underline{f}}_{a}=-1.5$, and ${\bar{f}}_{a}=1.5$. And, by calculation, $\underline{u}\leq \min\left(u\right)=-11.6763\leq u\leq 12.2333=\max\left(u\right)$; thus, for all above parameters, Assumption 3 can be satisfied. Meanwhile, for the simulation, we set $x\left(0\right)={\left[\begin{array}{cccc} 0.1 & -0.6 & 0.3 & -0.4 \end{array}\right]}^{T}$ and ${\hat{\zeta }}_{2}\left(0\right)={\left[\begin{array}{cc} 0.2104 & 2.6225 \end{array}\right]}^{T}$. We select $\Lambda =\left[\begin{array}{cc} -1.6 & 0\\ 0 & -0.8 \end{array}\right]$ which is a Metzler and Hurwitz matrix. The result shows that the interval estimation of $\overset{⌢}{y}$ is satisfactory. Finally, based on separation principle, we design $U=\left[\begin{array}{cccc} 27.8898 & -10.1000 & -3.4072 & 5.4474 \end{array}\right]$ such that the eigenvalues of A+BU are 4.3+5i,−4.3−5i,−5.5, and −1.

**Figure 1:**
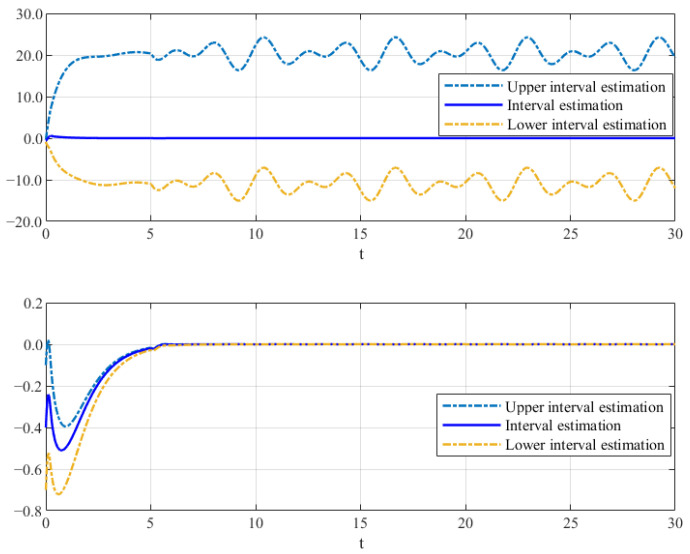
The interval estimation of $\overset{⌢}{y}$ by (16).

**Figure 2:**
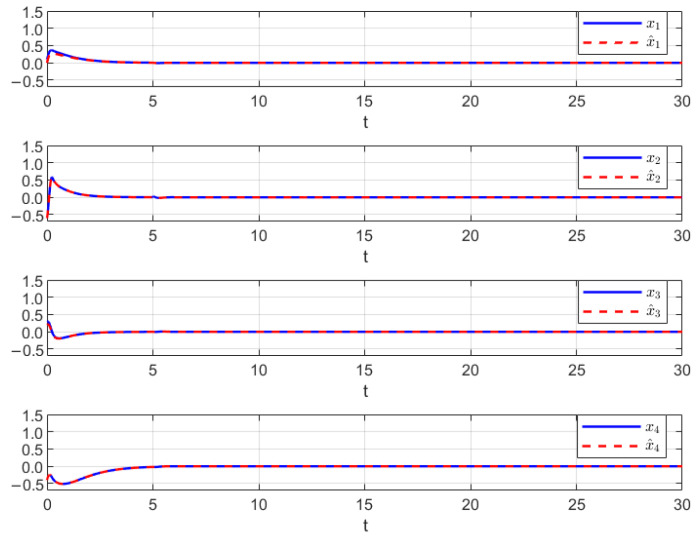
The state estimation by (12).

**Figure 3:**
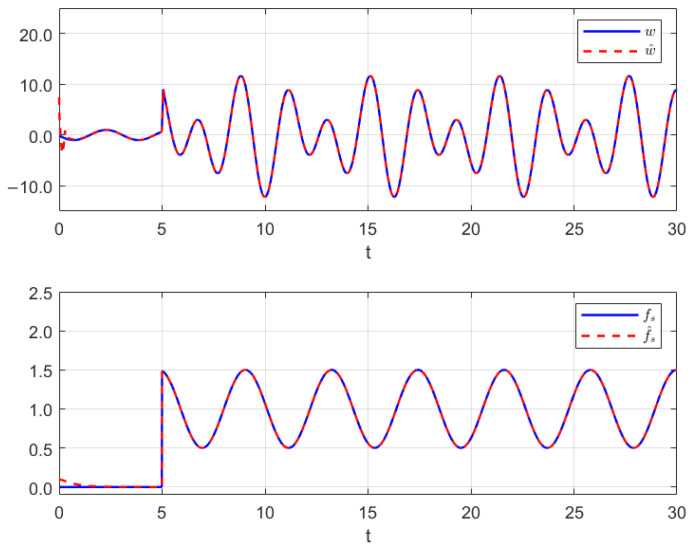
The MUIR by (25) and SFR through (12).

Thus, the effectiveness of the AFR-method-based reduced-order observer (12) and interval observer (16) can be validated by Figure 1, Figure 2 and Figure 3. Figure 1 shows that the interval observer (16) can offer the interval estimation of $\overset{⌢}{y}$ correctly. Figure 2 indicates that the reduced-order observer (12) can estimate the actual state *x* asymptotically. In Figure 3, the MUIR (25) can reconstruct the MUIs asymptotically, and the sensor fault $f_{s}\left(t\right)$ can be reconstructed by ${\hat{f}}_{s}$ effectively. Moreover, the FTC performance also reflected by Figure 2. From Figure 2, we can see that the system states are driven to zero under the FTC, even if the system has external disturbance for the actuator fault and sensor fault.

### 5.2. Example 2

**Figure 4:**
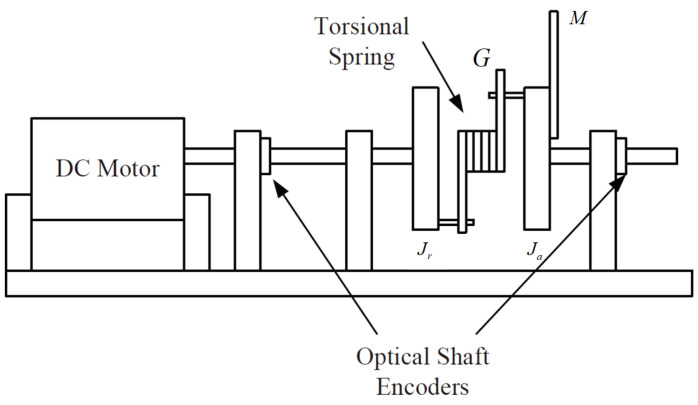
A single-link robotic arm.

We consider a single-link robotic arm with a rotary joint driven by an electric motor, as illustrated in Figure 4. The dynamics of the model can be described by [44]:

$$\begin{array}{c} \begin{array}{cc} \; & {\dot{\phi }}_{r}=\Omega_{r}\\ \; & {\dot{\Omega }}_{r}=\frac{\kappa }{J_{r}}\left(\phi_{a}-\phi_{r}\right)-\frac{C_{vd}}{J_{r}}\Omega_{r}+\frac{G}{J_{r}}u\\ \; & {\dot{\phi }}_{a}=\Omega_{a}\\ \; & {\dot{\Omega }}_{a}=-\frac{\kappa }{J_{a}}\left(\phi_{a}-\phi_{r}\right)-\frac{MgL}{J_{a}}\sin\left(\phi_{a}\right) \end{array} \end{array}$$

where $\Omega_{r}$ and $\Omega_{a}$ are, respectively, the angular velocities of the rotor and the arm; $\phi_{r}$ represents the angular rotation of the rotor; $\phi_{a}$ represents the angular position of the arm; $J_{r}$ and $J_{a}$ are the moment of inertia of the rotor and the arm, respectively; κ is the torsional stiffness coefficient; $C_{vd}$ is the damping coefficient; *G* is the driven gain; *u* is the control input signal; *g* stands for the gravitational acceleration; *M* is the payload mass; and *L* represents the moment arm length. Now, the state-space representation of the robotic system can be described by $\dot{x}=\bar{A}x+Bu+h\left(x\right)$, where

$$x=\left[\begin{array}{c} \phi_{r}\\ \Omega_{r}\\ \phi_{a}\\ \Omega_{a} \end{array}\right],\;\;\;\;A=\left[\begin{array}{cccc} 0 & 1 & 0 & 0\\ -\frac{\kappa }{J_{r}} & -\frac{C_{vd}}{J_{r}} & \frac{\kappa }{J_{r}} & 0\\ 0 & 0 & 0 & 1\\ \frac{\kappa }{J_{a}} & 0 & -\frac{\kappa }{J_{a}} & 0 \end{array}\right],\;\;\;\;B=\left[\begin{array}{c} 0\\ \frac{G}{J_{r}}\\ 0\\ 0 \end{array}\right],\;\;\;\;h\left(x\right)=\left[\begin{array}{c} 0\\ 0\\ 0\\ -\frac{MgL}{J_{a}}\sin\left(x_{3}\right) \end{array}\right],$$

where Assumptions 1 and 2 hold. Corresponding to the fault model, the system becomes

(33) $$\begin{array}{c} \left\{\begin{array}{cc} \dot{x} & =Ax+B\left(u_{f}+d\right)+h\left(x\right)\\ y & =Cx+Ff_{s} \end{array}\right. \end{array}$$

In order to apply our method to the nonlinear system (33), we linearize (33). As

$$\begin{array}{c} A_{0}={\left.\frac{\partial f}{\partial x}\right|}_{x=0}=\left[\begin{array}{cccc} 0 & 0 & 0 & 0\\ 0 & 0 & 0 & 0\\ 0 & 0 & 0 & 0\\ 0 & 0 & -\frac{MgL}{J_{a}} & 0, \end{array}\right] \end{array}$$

(33) is linearized in form (1) by setting

$$\begin{array}{c} A:=A+A_{0}=\left[\begin{array}{cccc} 0 & 1 & 0 & 0\\ -\frac{\kappa }{J_{r}} & -\frac{C_{vd}}{J_{r}} & \frac{\kappa }{J_{r}} & 0\\ 0 & 0 & 0 & 1\\ \frac{\kappa }{J_{a}} & 0 & -\frac{\kappa }{J_{a}}-\frac{MgL}{J_{a}} & 0 \end{array}\right] \end{array}$$

For the simulation, we select the parameters $\kappa =1.875,J_{r}=J_{a}=0.5,G=0.5$, $C_{vd}=7.5\times 10^{-4},g=9.8,L=0.1699$, and M=0.1. The matrices *C* and *F* are selected as

$$\begin{array}{c} C=\left[\begin{array}{cccc} 1 & 0 & 0 & 0\\ 0 & 1 & 0 & 1\\ 0 & 0 & 1 & 0 \end{array}\right],\;\;\;\;F=\left[\begin{array}{c} 1\\ 0\\ 0 \end{array}\right]. \end{array}$$

In addition, the disturbance is chosen as d=0.5sin(2t)+0.5cos(2t) and the faults are chosen as

$$\rho =\left\{\begin{array}{c} 0,t\leq \;5\\ 0.5,t>5 \end{array},\right.\;\;\;\;f_{a}=\left\{\begin{array}{c} 0.5\cos(3t),t\leq \;5\\ 1.5\sin(3t),t>5 \end{array},\right.\;\;\;\;f_{s}=\left\{\begin{array}{c} 0.2,t\leq \;5\\ 0.5\sin(1.5t-1)+1,t>5 \end{array}.\right.$$

Similar to Example 1, one can obtain that, by selecting the same Υ as Example 1,

$$\begin{array}{c} \tilde{C}=\left[\begin{array}{cccc} 0 & 0 & 0 & 0\\ 0 & 1 & 0 & 1\\ 0 & 0 & 1 & 0 \end{array}\right],\;\;\;\;\overset{⌢}{C}=\left[\begin{array}{cccc} 0 & 1 & 0 & 1\\ 0 & 0 & 1 & 0 \end{array}\right] \end{array}$$

Then, based on Smith orthogonal procedure, there exists a nonsingular matrix $S=\left[\begin{array}{cc} 1 & 0\\ 0 & 1 \end{array}\right]$ and $\overset{˘}{C}=\overset{⌢}{C}$ satisfying $\overset{⌢}{C}=S\overset{˘}{C}$, where $\overset{˘}{C}\in R^{\hat{p}\times n}$ and ${\overset{˘}{C}}^{T}=I_{\hat{p}}$. We extend matrix $\overset{˘}{C}$ to be an n×n orthogonal matrix:

$$\begin{array}{c} W=\left[\begin{array}{cccc} 0 & 0.7071 & 0 & 0.7071\\ 0 & 0 & 1 & 0\\ 0.7071 & -0.5 & 0 & 0.5\\ -0.7071 & -0.5 & 0 & 0.5 \end{array}\right]. \end{array}$$

Now, solving (8) gives

$$\begin{array}{cc} P & =10^{4}\times \left[\begin{array}{cccc} 1.8091 & -0.0000 & -0.2687 & -0.1053\\ -0.0000 & 0.0001 & 0.0000 & 0.0001\\ -0.2687 & 0.0000 & 1.4791 & -0.4856\\ -0.1053 & 0.0001 & -0.4856 & 0.5853 \end{array}\right],\\ K & =10^{3}\times \left[\begin{array}{cc} 0.0009 & -0.0005\\ 2.5673 & 0.9198\\ -0.0004 & 0.0016\\ -0.0010 & 0.0002 \end{array}\right],H=\left[\begin{array}{cc} 1.3491 & 0.0152 \end{array}\right]. \end{array}$$

Thus, we have

$$\begin{array}{cc} \bar{A} & =\left[\begin{array}{cccc} -0.0008 & -0.2355 & 0.0005 & 0.0005\\ 0.7071 & 0.0000 & 0.5000 & 0.5000\\ 0.5005 & -3.9165 & 2.2977 & -3.0056\\ -0.4995 & -3.9165 & 3.0048 & -2.2985 \end{array}\right],\\ \bar{B} & =\left[\begin{array}{c} 0.7071\\ 0.0000\\ -0.5000\\ -0.5000 \end{array}\right],\bar{C}=\left[\begin{array}{cccc} 1.4142 & 0.0000 & -0.0000 & 0.0000\\ 0.0000 & 1.0000 & 0.0000 & 0.0000 \end{array}\right],\\ \bar{P} & =10^{4}\times \left[\begin{array}{cccc} 0.2928 & -0.3434 & 0.1542 & 0.2595\\ -0.3434 & 1.4791 & -0.4328 & -0.0528\\ 0.1542 & -0.4328 & 0.9764 & -0.7583\\ 0.2595 & -0.0528 & -0.7583 & 1.1253 \end{array}\right],\\ \bar{K} & =10^{3}\times \left[\begin{array}{cc} 1.8146 & 0.6505\\ -0.0004 & 0.0016\\ -1.2835 & -0.4602\\ -1.2848 & -0.4595 \end{array}\right]. \end{array}$$

Given ${\bar{A}}_{11}=\left[\begin{array}{cc} -0.0008 & -0.2355\\ 0.7071 & 0.0000 \end{array}\right]$, ${\bar{A}}_{21}=\left[\begin{array}{cc} 0.5005 & -3.9165\\ -0.4995 & -3.9165 \end{array}\right]$, ${\bar{A}}_{12}=\left[\begin{array}{cc} 0.0005 & 0.0005\\ 0.0005 & 0.0005 \end{array}\right]$, ${\bar{A}}_{22}=\left[\begin{array}{cc} 2.2977 & -3.0056\\ 3.0048 & -2.2985 \end{array}\right]$, ${\bar{P}}_{2}=10^{4}\times \left[\begin{array}{cc} 0.1542 & -0.4328\\ 0.2595 & -0.0528 \end{array}\right]$, ${\bar{P}}_{3}=10^{4}\times \left[\begin{array}{cc} 0.9764 & -0.7583\\ -0.7583 & 1.1253 \end{array}\right]$, we can calculate $\bar{L}=\left[\begin{array}{cc} 0.7071 & -1.0063\\ 0.7071 & -0.7250 \end{array}\right]$, ${\overset{˘}{A}}_{2}=\left[\begin{array}{cc} 1.0000 & -4.8203\\ -1.0000 & -3.3526 \end{array}\right]$. Finally, based on these matrix parameters, the reduced-order observer (12) can be constructed.

**Figure 5:**
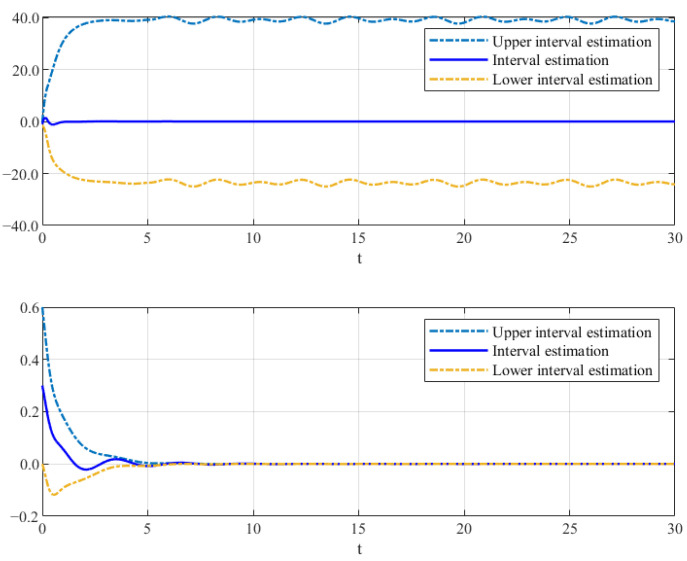
The interval estimation of $\overset{⌢}{y}$ by (16).

We select $\underline{u}=-25$ and $\bar{u}=35$, and the resting initial values and some parameters are selected the same as Example 1, where Assumption 3 holds. For the interval observer (16), we select a Metzler and Hurwitz matrix $\Lambda =\left[\begin{array}{cc} -1.6 & 0\\ 0 & -0.8 \end{array}\right]$. The output interval estimation performance is given in Figure 5. Based on the separation principle, we determine the feedback gain *U* by the pole place method: U=[−97.0570−15.098542.2233−71.5551], which can result in the eigenvalues of A+BU being −5.5,−1, and 4.3±5i.

**Figure 6:**
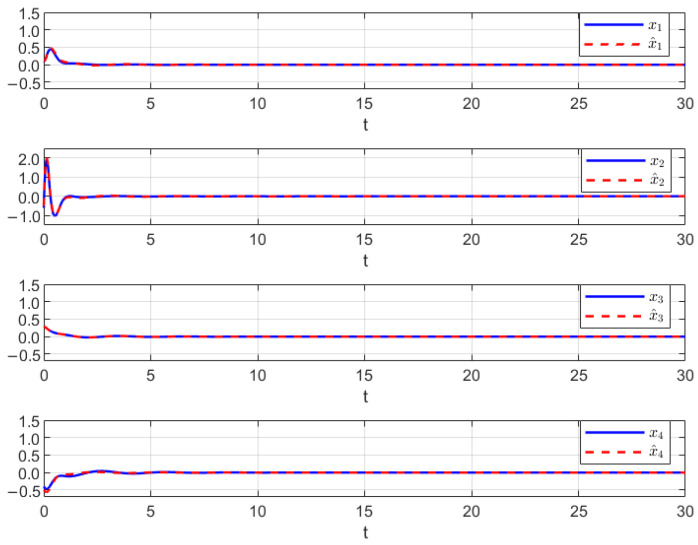
The state estimation by (12).

**Figure 7:**
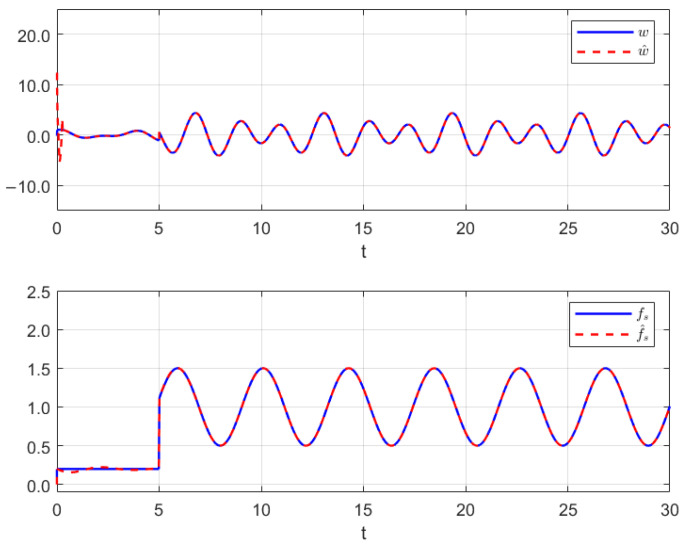
The MUIR by (25) and SFR through (12).

The interval observer (16) provides correct interval estimation in Figure 5, while the reduced-order observer (12) achieves asymptotic convergence state estimation in Figure 6. Figure 7 shows the performances of the MUIR and SFR. The FTC performance is also reflected by Figure 6, and it demonstrates that the system’s asymptotical stability can be reached although the system has an external disturbance, the actuator fault sensor fault.

**Remark** **4.** *Based on some of the existing literature [35,37], which investigates FTC problems, the primary advantages of the proposed method in the present paper can be summarized as follows: First, unlike most existing studies that only address either actuator faults or sensor faults separately, our approach simultaneously handles both sensor and actuator faults in the presence of external disturbances, offering a more comprehensive fault-tolerant control solution for complex uncertain systems. Second, instead of relying on robust or adaptive control techniques, which typically ensure only bounded convergence, we propose a novel MUIR method that asymptotically estimates the compounded actuator and disturbance signals. Then, by introducing the MUIR into the controller, a compensation controller is designed, leading to asymptotic stability of the closed-loop system, rather than merely bounded-error performance. Third, the proposed observer-based fault-tolerant control scheme satisfies the separation principle, meaning that the observer design, fault reconstruction, and controller synthesis can be carried out independently, thereby simplifying the overall design process and enhancing practical applicability.*

## 6. Conclusions

In conclusion, this paper proposes a novel reduced-order observer-based approach for FR and FTC for systems with disturbance, actuator, and sensor faults. By introducing an output transformation to decouple sensor faults, an equivalent sensor fault-free system is derived. To address the unmeasured system state variables, actuator faults, and external disturbances, a reduced-order observer is designed first, and then an MUIR method is proposed through an interval observer. Subsequently, an FTC scheme is further developed to ensure system stability in the presence of faults. Theoretical analysis confirms that asymptotic stability of the uncertain systems can be guaranteed under the proposed observer-based FTC scheme. It should be emphasized that the designs of the reduced-order observer, the MUIR, and the FTC meet the separation principle. Determining how to introduce some new techniques, such as even-triggered strategy, to improve the proposed methods will be our further consideration. 

## Figures and Tables

**Table 1:** Common fault types.

Fault Mode	$\underline{\varrho }$	$\bar{\varrho }$	$f_{a}\left(t\right)$
Normal	1	1	$0_{m}$
Outage	0	0	$0_{m}$
Bias	1	1	≠$0_{m}$
Stuck	0	0	≠$0_{m}$
Loss performance	>0	<1	$0_{m}$

## Data Availability

The original contributions presented in this study are included in the article. Further inquiries can be directed to the corresponding author.
